# Metabolomics and lipid profile analysis of *Coccomyxa melkonianii* SCCA 048

**DOI:** 10.1007/s00792-021-01234-z

**Published:** 2021-05-31

**Authors:** Giacomo Fais, Veronica Malavasi, Paola Scano, Santina Soru, Pierluigi Caboni, Giacomo Cao

**Affiliations:** 1 Interdepartmental Centre of Environmental Science and Engineering (CINSA), University of Cagliari, via San Giorgio 12, 09124 Cagliari, Italy; 2 Department of Life and Environmental Sciences, University of Cagliari, 09124 Cagliari, Italy; 3 Department of Mechanical, Chemical and Materials Engineering, University of Cagliari, piazza d’Armi, 09123 Cagliari, Italy

**Keywords:** GC–MS, LC–MS, Green algae, Phycoremediation, SQDG, FAHFA

## Abstract

With an unsupervised GC–MS metabolomics approach, polar metabolite changes of the microalgae *Coccomyxa melkonianii* SCCA 048 grown under standard conditions for seven weeks were studied. *C. melkonianii* was sampled at the Rio Irvi River, in the mining site of Montevecchio-Ingurtosu (Sardinia, Italy), which is severely contaminated by heavy metals and shows high concentrations of sulfates. The partial-least-square (PLS) analysis of the GC–MS data indicated that growth of *C. melkonianii* was characterized by an increase of the levels of threonic acid, *myo*-inositol, malic acid, and fumaric acid. Furthermore, at the sixth week of exponential phase the lipid fingerprint of *C. melkonianii* was studied by LC-QTOF-MS. *C. melkonianii* lipid extract characterized through an iterative MS/MS analysis showed the following percent levels: 61.34 ± 0.60% for triacylglycerols (TAG); 11.55 ± 0.09% for diacylglyceryltrimethyl homoserines (DGTS), 11.34 ± 0.10% for sulfoquinovosyldiacylglycerols (SQDG) and, 5.29 ± 0.04% for lysodiacylglyceryltrimethyl homoserines (LDGTS). Noteworthy, we were able to annotate different fatty acid ester of hydroxyl fatty acid, such as FAHFA (18:1_20:3), FAHFA (18:2_20:4), FAHFA (18:0_20:2), and FAHFA (18:1_18:0), with relevant biological activity. These approaches can be useful to study the biochemistry of this extremophile algae in the view of its potential exploitation in the phycoremediation of polluted mining areas.

## Introduction

Microalgae belonging to the class of Trebouxiophyceae can be found in different terrestrial and aquatic environments, such as mesophilic habitats comprising soil, salty waters and extreme environments (Büdel et al. 2009; Cavacini 2001; Fermani et al. 2007; Flechtner et al. 2013; Heesch et al. 2012; Hodač et al. 2016; Juárez et al. 2011; Malavasi et al. 2016; Tragin and Vaulot 2018).

Trebouxiophyceae show different phenotypic, physiologic, and genetic characteristics determining their ability to survive in these environments (Assunção et al. 2017; Cannell 1993). Belonging to this class,* Coccomyxa* spp. with more than 60 described species (Guiry et al. 2020) are morphological characterized by a parietal chloroplast without a pyrenoid and a thin three-layered cell wall, and by the lack of flagellum (Brunner and Honegger 1981; Darienko et al. 2015; Malavasi et al. 2016). In recent years, in response to the search for more biofuel sources, strains of the genus *Coccomyxa* have been studied for their ability to produce lipids and, given the high concentration of polyunsaturated ω-3 and ω-6 fatty acids, some strains may cover an important role in the food and feed industry (Jeong et al. 2011). Moreover, green algal photobionts of *Coccomyxa* are often symbiotic partners in Peltigera lichens which are relatively sensitive to environment while growing exclusively in mild moist habitats (Guschina and Harwood 2006). These species can also resist in strongly polluted environments (Kalinowska and Pawlik-Skowrońska 2010; Koechler et al. 2016; la Rocca et al. 2009).

In our study, the extremophile microalga *C. melkioananii* SCCA 048 was sampled in acidic mine drainage waters severely contaminated by heavy metals. This mining site of Montevecchio-Ingurtosu (Sardinia, Italy) is characterized by minerals such as galena (PbS_2_) containing appreciable levels of silver, sphalerite ((Zn, Fe)S) containing Cd, Ga, In, and the oxide mineral goethite (α-FeO(OH)). For this reason, this site is rich in pollutants, with levels of Zn^2+^ at 956 mg/L, Fe^2+^ at 227 mg/L, and sulfates at 3697 mg/L, released into the river by oxidation reactions involving the sulfide minerals still present in the ore bodies after flooding of galleries (De Giudici et al. 2018).

This *C. melkonianii* strain well adapted to this contaminated environment may be used for the development of new and sustainable phycoremediation technology strategies (De Giudici et al. 2018; Kothe et al. 2005). Furthermore, *Coccomyxa actinabiotis* showed high silver levels confined inside microalgae, when grown in contaminated waters containing silver ions (Leonardo et al. 2016). Additionally, this species isolated from a nuclear facility showed the ability to uptake radionuclides (Rivasseau et al. 2016; Sukla and Pradhan 2019). In this context, *Coccomyxa subellipsoidea*, a unicellular green acidophilic microalga isolated from the Antarctic, grows over a range of temperate climates, accumulating high levels of triglycerides under abiotic stress conditions and thus relevant for biofuel/bioproduct production, or for providing biomass for nitrogenous biofertilizers, and for the degradation of organophosphate pesticides (Allen et al. 2015; Heesch et al. 2012; Hirooka et al. 2017; Nicodemus et al. 2020). It is also known that *Coccomyxa* species can produce lipid-like compounds containing a dimethylarsinoyl group cultivated at high concentration of disodium hydrogen arsenate (Řezanka et al. 2019). Furthermore, this strain can be exploited for the production of isotopically labeled (non-radioactive) lipids to be used in the field of analytical chemistry (Beherens 1994).

In our previous studies, this strain was investigated for its ability of growing at different pH values. Our results demonstrated the extreme plasticity of this species, being able to cope with different environments and to produce more than 20% by weight of lipids (Soru et al. 2019a). Interestingly, a change in the profile of fatty acid methyl esters (FAMEs) and an increase in lipid content under nitrogen starvation was also observed, showing that *C. melkonianii* SCCA 048 can be used for biofuels production (Soru et al. 2019b).

Metabolomics is as a new “omics” science widely used in system biology. Metabolomics is the holistic study of the metabolome of a system cell, tissue, or organism performed using specific analytical instrumentation and statistical methods. The metabolome and lipidome are the result of the interaction of the genome of the system with its environment (Rochfort 2005) and comprise the collection of all low molecular weight compounds, such as amino acids, carbohydrates, nucleotides, organic acids, fatty acids, lipids, and cofactors responsible for maintaining cell's biological processes (Dunn and Ellis 2005). Metabolomics and lipidomics studies play an important role to understand the physiological changes in living organisms, thus offering additional insight for microalgae strain engineering consideration (Arora et al. 2018; Ito et al. 2013). Several metabolomics studies evidenced that the production of metabolites is highly dependent on the development of the microalgal cells (Blifernez-Klassen et al. 2018; Treves et al. 2017; Vidoudez and Pohnert 2012) and omics approach has been used to characterize compounds synthesized during lipids accumulation (Arora et al., 2018). Synthesis of microalgae metabolites is tighten correlated to the accumulation of triacylglycerols (TAGs) during nitrogen starvation and the presence of specific compounds. For example, an increase of Krebs cycle metabolites, such as citrate, 2-oxoglutarate, and phosphorylated sugars, with a concomitant decreases in amino acids levels indicates an accumulation of intracellular lipids (Blaby et al. 2013; Chen et al. 2017; Ito et al. 2013; Wase et al. 2014). Similarly, cell metabolites, such as glycerol, 3-phosphoglyceric acid and 2-ketoglutaric acid, play a crucial role in the increase of TAGs in response to environmental stress factors, such as high salinity, high intensity of light or to chemical challenge with phytohormones (Ho et al. 2015, 2014; Malavasi and Cao 2015; Yu et al. 2016). Furthermore, polar lipids, i.e. phosphatidic acid (PA), phosphatidylcholine (PC), phosphatidylethanolamine (PE), phosphatidylglycerol (PG), digalactosyldiacylglycerol (DGDG), monogalactosyldiacylglycerol (MGDG), sulphoquinovosyl diacylglycerols (SQDG), and diacylglyceryltrimethylhomoserine (DGTS), play an important role in membranes structure, photosynthesis, energy storage and cellular signaling (Darienko et al., 2015; Van Meer et al., 2008).

Despite its physiological and technological importance, little is known on the metabolome and lipidome of *C. melkonianii* SCCA 048 (Pasqualetti et al. 2015; Soru et al. 2019a, b).

Microalgae contain large amounts of lipids, proteins, and carbohydrates, while one of the main bottlenecks for their possible application is that the composition of such compounds dramatically changes during growth depending upon the relevant species and strains (Guedes et al. 2011; Cannell 1993; De Morais et al. 2015). It is also highly desirable to take advantage of suitable microalgal species which can be exploited for the isolation of high-value metabolites (Lee et al. 2018) and lipids as well as potent and ecofriendly tools for bioremediation of polluted waters.

To understand the molecular profiling and to evaluate the changes occurring during the growth of this microalga, we employed an untargeted metabolomic approach.

In this longitudinal study, the changes of cellular metabolites of *Coccomyxa melkonianii* strain SCCA 048 during seven weeks of cultivation, were studied using a gas chromatography mass spectrometry (GC–MS) approach followed by a partial-least-square analysis (PLS). Furthermore, using a UHPLC-QTOF-MS iterative approach we studied the lipidome fingerprint of *C. melkonianii.* The aim of this study was to explore the metabolic profiles of *C. melkonianii* SCCA 048 grown under standard conditions, and to obtain basic lipidome profile information for the potential use of this microalga in the bioremediation of the drainage waters of the Rio Irvi insisting in the mining area of Ingurtosu.

## Materials and methods

### Strains, culture conditions and growth measurement

**Fig. 1:**
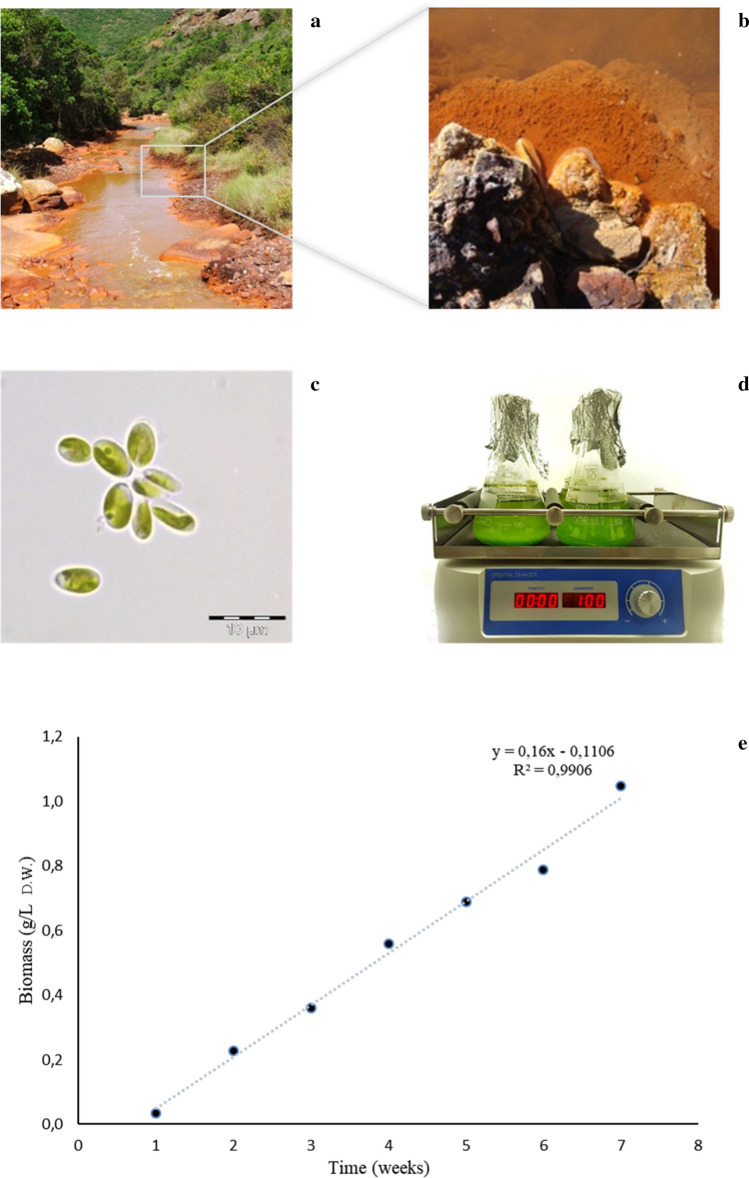
**a** and **b** habitat of *Coccomyxa melkonianii* SCCA 048, Rio Irvi SW Sardinia (Italy); **c** cells of *C. melkonianii* (Scale bar: 10 μm); **d** strain cultured for 7 weeks; **e** time evolution of biomass concentration (g/L DW)

In this work, freshwater strains of *C.* melkonianii SCCA 048, sampled at the Rio Irvi river (West-Sardinia, Italy), was investigated (Fig. 1b). The strain was maintained under axenic conditions at the Sardinian Culture Collection of Algae (SCCA) (Malavasi and Cao 2015). The alga strain was phototrophically cultivated at 25 °C under 12:12 light–dark illumination of 60–80 μmol photons/m^2^/s (Light meter Delta) white light and continuous agitation at 100 rpm (Stuart SSM1, Biosigma orbital shaker) in BBM medium for 49 days. The cultivation was performed in cylindrical Erlenmeyer flasks (with maximal capacity of 250 mL) with a total starting volume of 150 mL per replicate. Flasks as well as the culture media were autoclaved at 121 °C for 15 min prior to microalgae inoculation. Flasks were stoppered by cotton plugs wrapped in cotton gauze during cultivation. All operations were conducted under a microbiological safety cabinet. A pre-culture of 5 days was prepared as inoculum for the experiments and used when cells started their exponential growth. Such pre-culture was incubated and maintained under the same conditions described above. The microalgae growth was monitored in vivo by detecting the chlorophyll-a optical density (OD) of the culture at 663 nm (Genesys 20. Thermo Scientific. Waltham. USA). Cells morphology was investigated using an inverted light microscope (Olympus, Tokyo, Japan).

### Chemicals

Chemicals and solvents were purchased from (Sigma Aldrich, Milano, Italy). Bi-distilled water was obtained with a MilliQ purification system (Millipore, Milan, Italy). Methoxyamine hydrochloride, NO-bis (trimethylsilyl) trifluoroacetamide (BSTFA) were used for the derivatization process. A SPLASH^®^ LIPIDOMIX^®^ standard component mixture was purchased from Sigma Aldrich (Milan, Italy) PC (15:0–18:1) (d7), PE(15:0–18:1) (d7), PS (15:0–18:1) (d7), PG (15:0–18:1) (d7), PI (15:0–18:1) (d7), PA (15:0–18:1) (d7), LPC (18:1) (d7) LPC 25, LPE (18:1) (d7), Chol Ester (18:1) (d7), MG (18:1) (d7), DAG (15:0–18:1) (d7), TAG ((15:0–18:1) (d7)-15:0)), SM (18:1) (d9), Cholesterol (d7).

### GC–MS analysis

For GC–MS analysis, 10 mL of culture was taken at seven different time points (1, 7, 15, 22, 29, 36, and 43 days) of the cultivation and transferred into a sterile 15 mL Falcon tube. Samples were then stored at -20 °C. After thawing, microalgae samples were sonicated for 15 min at 4 °C at 13,000 rpm. Samples were vortexed for 30 s and 250 μL from each sample was transferred into Eppendorf tubes, to which 250 μL of methanol and 125 μL of chloroform (2:1 *v/v*) were subsequently added (Folch et al. 1987). Samples were stored at room temperature, vortexed every 15 min and then centrifuged for 10 min at 13,000 rpm at 4 °C. The supernatant was separated, dried under nitrogen flow and derivatized with 50 μL of methoxyamine/pyridine solution (10 mg/mL). After 17 h, 50 μL of BSTFA were added for 40 min at 50 °C. Samples were re-suspended with 50 μL of hexane. After derivatization, samples were injected in a Hewlett Packard 6850 Gas Chromatograph, 5973 mass selective detector (Agilent Technologies, Palo Alto, CA), using helium as carrier gas at 1.0 mL/min flow. 1 μL of each sample was injected in the split-less mode and resolved on a 30 m × 0.25 mm × 0.25 μm DB-5MS column (Agilent Technologies, Palo Alto, CA). Inlet, interface, and ion source temperatures were 250, 250 and 230 °C, respectively. Oven starting temperature was set to 50 °C, final temperature to 230 °C with a heating rate of 5 °C/min for 36 min and then for 2 min at a constant temperature. Electron impact mass spectra were recorded from m/z 50 to 550 at 70 eV. Chromatograms in the AIA format were then uploaded to the XCMS Online platform (Tautenhahn et al. 2012). The outputs of XCMS consisted of a list of features corresponding to the intensity value of each m/z ion at a specific retention time value. The identification of metabolites was performed by mass spectra comparison with analytical standards, using the NIST14 library database of the National Institute of Standards and Technology (Gaithersburg, MD), Golm library (http://gmd.mpimp-golm.mpg.de/), and an in-house library of metabolites.

### Multivariate analysis (MVA)

The GC–MS data were submitted to MVA as implemented in SIMCA-P + software (version 14.1. Umetrics, Umeå, Sweden). Prior to MVA, GC features were mean centered and scaled to unit variance column-wise. Principal component analysis (PCA) was performed to investigate sample distributions, deviating features and prevailing trends. GC–MS data were correlated to the calculated biomass concentration (g/L) by a single-Y Partial Least Squares Projections to Latent Structures (PLS) analysis and to highlight the metabolites mostly correlated to the time evolution of biomass by its Orthogonal variant (OPLS). The quality of the model was evaluated based on the cumulative parameters *R*^2^*Y* and *Q*^2^*Y*, being the latter estimated by the default leave-1/7th-out cross-validation. The variable influence on projection (VIP) scores, that summarize the contribution of each variable to the model, were analyzed (Scano et al. 2014). GC–MS features showing VIP values > 1 underwent a manual annotation using GC–MS library mass spectral databases. A metabolite was considered significant only when at least two of its most abundant mass fragments and a retention index deviation < 0.05 min were found in the list of VIP having a score greater than 1. For quantification purposes for each metabolite, we considered the intensity of the most abundant mass fragment.

### UHPLC-QTOF-MS/MS analysis

The chloroform layer of the sample at the sixth week of the exponential phase was obtained by Folch extraction (Folch et al., 1987) was evaporated under a gentle nitrogen stream, and dissolved in 100 µL of a mixture of acetonitrile/water (1:1 *v/v*) and 10 µL of a mixture of methanol/chloroform (1:1 *v/v*), and finally added of 10 µL of the internal lipid standard SPLASH solution. Samples were then analyzed with a LC-QTOF-MS coupled with an Agilent 1290 Infinity II LC system. An aliquot of 1.0 μL from each sample was injected in a Kinetex 5 µm EVO C18 100 A, 150 mm × 2.1 μm column (Agilent Technologies, Palo Alto, CA). The column was maintained at 50 °C at a flow rate of 0.4 mL/min. The mobile phase for positive ionization mode consisted of (A) 10 mM ammonium formate solution in 60% of milliQ water and 40% of acetonitrile and (B) 10 mM ammonium formate solution containing 90% of isopropanol, 10% of acetonitrile. In positive ionization mode, the chromatographic separation was obtained with the following gradient: initially 60% of A, then a linear decrease from 60 to 50% of A in 2 min then at 1% in 5 min staying at this percentage for 1.9 min and then brought back to the initial conditions in 1 min. The mobile phase for negative ionization mode differed only for the use of 10 mM ammonium acetate instead of ammonium formate. We used an Agilent jet stream technology source which was operated in both positive and negative ion modes with the following parameters: gas temperature, 200 °C; gas flow (nitrogen) 10 L/min; nebulizer gas (nitrogen), 50 psig; sheath gas temperature, 300 °C; sheath gas flow, 12 L/min; capillary voltage 3500 V for positive and 3000 V for negative; nozzle voltage 0 V; fragmentor 150 V; skimmer 65 V, octapole RF 7550 V; mass range, 50 − 1700 m/z; capillary voltage, 3,5 kV; collision energy 20 eV in positive and 25 eV in negative mode, mass precursor per cycle = 3; threshold for MS/MS 5000 counts. Before the analysis, the instrument was calibrated using an Agilent tuning solution at the mass range of m/z 50–1700. Samples were acquired in an auto MS/MS method in the iterative mode with a mass error tolerance of 20 ppm with a retention exclusion tolerance of 0.2 min. The Agilent MassHunter LC/MS Acquisition console (revision B.09.00) and Lipid annotator from the MassHunter suite was used for data acquisition and data processing. This method consists in injecting the same sample multiple times, while precursors previously selected for MS/MS fragmentation are excluded on a rolling basis. Five different iterative analyses were performed for maximizing the maximum number of lipid species detected. In the positive mode, lipids were quantified using the following standards TAG 15:0–18:1 (d7)-15:0, PC 15:0–18:1 (d7), PG 15:0–18:1 (d7), and PE 15:0–18:1 (d7). Microalgae lipid analysis was conducted using an innovative iterative auto MS/MS mode.

## Results and discussion

### Growth evaluation

As a first step towards the metabolic profiling of *C. melkonianii* SCCA 048, we monitored the growth of the microalgal strain along seven weeks. The experiments started (day 1) from biomass concentration lower than 0.1 g/L and showed a continuous increase in the time course of the cultivation with an almost linear growth pattern during the investigated period. At the end of cultivation, *C. melkonianii* achieved a final biomass concentration of approximately 1.05 g/L. The time evolution of biomass during cultivation is reported in Fig. 1e.

### GC–MS metabolomics

**Fig. 2:**
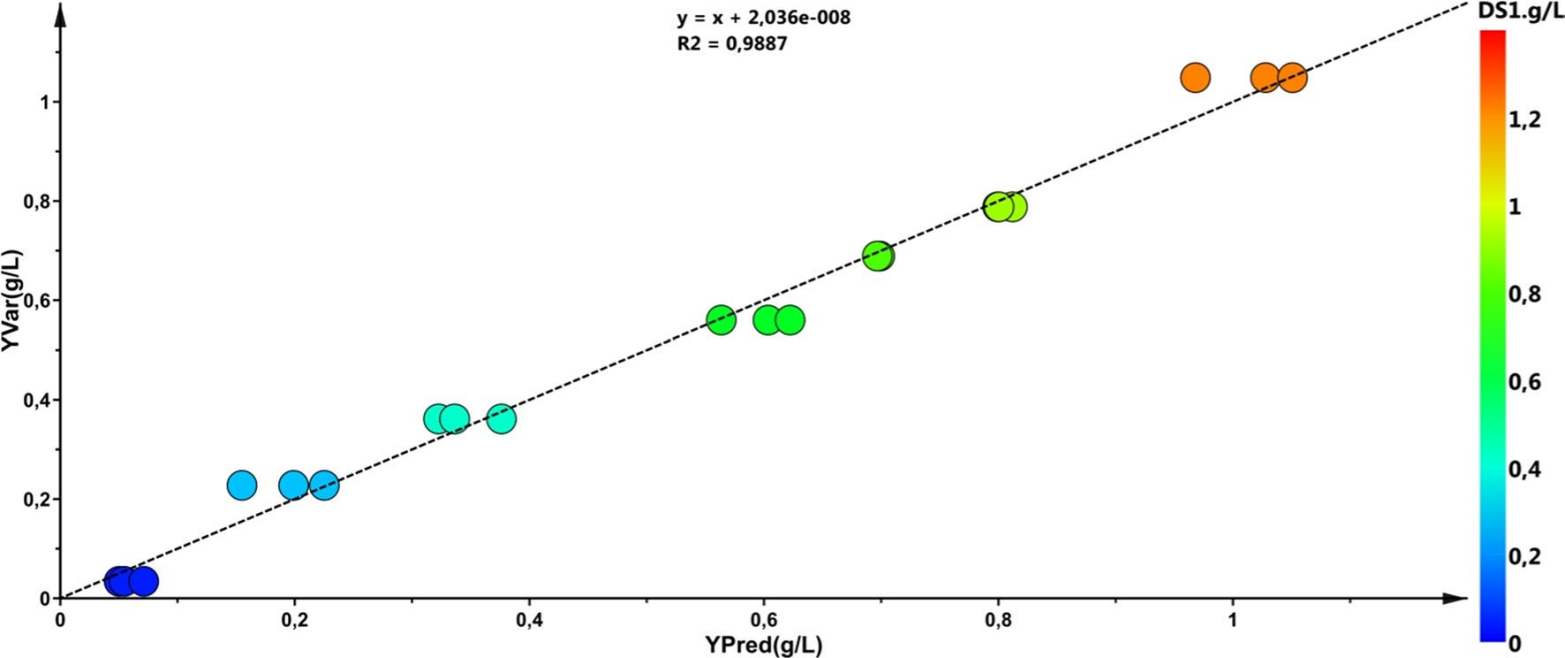
OPLS correlation plot between experimental (*y*-axis) and predicted (*x*-axis) biomass of *Coccomyxa melkonianii* SCCA 048 (*R*^2^*Y* = 0.99, *Q*^2^*Y* = 0.96)

GC–MS chromatograms of these alga were analyzed, 53 polar metabolites were detected, 39 of which were identified (Table 1). The GC–MS data, obtained by the XCMS pipeline, underwent multivariate analysis. To identify those metabolites mostly correlated with the growth of the culture of *C. melkonianii*, the OPLS predictive statistical analysis was applied. Results of the analysis, shown as correlation plot in Fig. 2, indicated that for this alga, the whole metabolite profile linearly changes with the biomass increase. Those metabolites with a variable influence on projection (VIP) values > 1 that increased during growth are reported in Table 2. Among these, threonic acid was found correlated with algae growth. Threonic acid is a storage metabolite of the ascorbate metabolism pathway. This metabolite is strongly elevated under stress conditions, playing a part as a stress-responsive factor with a bio-protective role, and thus helping the cells to retain cellular integrity and stability (Blifernez-Klassen et al. 2018). Several studies suggest that ascorbate efflux plays a role in Fe^3+^ reduction in plants and algae (Kobayashi and Nishizawa 2012; Urzica et al. 2012; Grillet et al. 2014; Smirnoff 2018). Levels of threonic acid as housekeeping metabolite in *C. melkonianii* might correlate with its adaptation to harsh habitats (Malavasi et al. 2016). Moreover, malic acid and citric acid that are time-correlated with the growth of *C. melkonianii* are also known to be able to chelate metals (Perpetuo et al., 2011; Asemave 2018) Furthermore, *myo*-inositol is required for the cell growth and development, being involved in the phosphatidylinositol signaling pathway that takes part in auxin transport, cell wall biosynthesis, phytic acid biosynthesis, and the production of stress-related compounds (Cho et al. 2015).

**Table 1:** GC–MS characteristics of *C. melkonianii* polar metabolites

	Metabolites	Rt (min)^a^	*m/z*	%	*m/z*	%	*m/z*	%
1	α-Hydroxybutyric acid (2 TMS)	16.08	131	100	147	86	73	78
2	Hexanoic acid (TMS)	16.16	75	100	73	90	117	25
3	Glycolic acid (2 TMS)	16.29	147	100	73	12	75	29
4	Alanine (2 TMS)	16.81	116	100	73	54	147	25
5	N-Acetyl-L-Alanine (TMS)	16.94	188	100	73	79	75	37
6	Unknown #1	17.19	73	100	258	90	116	45
7	Oxalic acid (2 TMS)	17.37	147	100	73	83	148	16
8	Unknown #2	17.51	147	100	73	89	133	41
9	Succinic acid (2 TMS)	17.70	73	100	147	53	89	29
10	β-Hydroxybutyric acid (2 TMS)	17.84	147	100	73	56	117	41
11	Unknown #3	18.29	73	100	132	90	75	28
12	Unknown #4	18.36	75	100	63	61	159	35
13	Valine (2 TMS)	18.77	144	100	73	45	218	25
14	Unknown #5	18.85	73	100	75	68	147	37
15	Butanoic acid (2 TMS)	19.02	147	100	75	64	73	42
16	Unknown #6	19.06	73	100	147	90	75	63
17	Unknown #7	19.16	73	100	75	42	100	50
18	Unknown #8	19.35	73	100	75	73	147	47
19	Serine (2 TMS)	19.39	75	100	132	57	116	54
20	Aminoethanol (TMS)	19.54	174	100	73	42	175	18
21	Phosphoric acid (3 TMS)	19.68	299	100	300	46	314	35
22	Glycine (3 TMS)	20.15	174	100	73	54	147	29
23	Succinic acid (2 TMS)	20.19	147	100	73	45	75	27
24	Glyceric acid (3 TMS)	20.52	73	100	147	56	292	416
25	Fumaric acid (2 TMS)	20.63	245	100	73	46	147	42
26	Unknown #9	20.74	73	100	75	65	147	41
27	Nonanoic acid (3 TMS)	20.79	75	100	73	96	215	77
28	Unknown #10	21.29	73	100	75	57	213	45
29	Unknown #11	21.55	73	100	75	48	147	44
30	Unknown #12	21.70	73	100	75	83	37	81
31	Unknown #13	21.74	73	100	75	76	160	47
32	Decanoic acid (3 TMS)	22.09	75	100	73	79	229	71
33	Aminomalonic acid (3 TMS)	22.40	218	66	320	31	133	21
34	Malic acid (3 TMS)	22.63	233	25	245	16	148	11
35	Adipic acid (2 TMS)	22.73	73	100	75	736	111	41
36	Pyroglutamic acid (TMS)	23.03	156	100	73	56	75	23
37	γ-Aminobutyric acid (3 TMS)	23.10	174	100	354	30	175	18
38	Erythronic acid (4 TMS)	23.39	73	100	147	54	292	47
39	Threonic acid (4 TMS)	23.60	73	100	292	60	147	54
40	Glutaric acid (2 TMS)	23.69	73	100	75	41	147	38
41	Glutamic acid (3 TMS)	24.22	246	100	73	42	247	23
42	Lauric acid (TMS)	24.48	257	100	73	94	75	85
43	Phosphoric acid (4 TMS)	25.89	357	100	299	93	73	82
44	Azelaic acid (2 TMS)	26.11	73	100	75	71	317	32
45	Propanoic acid (4 TMS)	26.39	73	100	357	73	299	72
46	Citric acid (4 TMS)	26.50	465	11	273	90	375	19
47	Myristic acid (TMS)	26.60	73	100	285	80	75	66
48	Unknown #14	26.94	242	100	73	47	27	33
49	Gluconic acid (6 TMS)	28.47	73	100	333	61	292	58
50	Palmitic acid (TMS)	28.53	313	100	117	67	73	62
*51*	*Myo*-Inositol (6 TMS)	29.29	305	100	217	81	73	79
52	Stearic acid (3 TMS)	30.30	341	100	117	61	73	66
53	Gluconic acid (4 TMS)	32.26	73	100	387	73	299	63

^a^Retention time (min)

**Table 2:** OPLS metabolites positively correlated with growth of *Coccomyxa melkonianii* SCCA

Metabolites	Rt (min)^a^	VIP^b^
Threonic acid (4 TMS^c^)	23.59	1.48
*Myo*-inositol (6 TMS)	29.28	1.48
Malic acid (3 TMS)	22.63	1.46
Fumaric acid (2 TMS)	20.64	1.42
Phosphoric acid (4 TMS)	30.15	1.37
Citric acid (4 TMS)	26.50	1.35
Succinic acid (2 TMS)	20.19	1.34
Propanoic acid (4 TMS)	26.39	1.33
β-Hydroxybutyric acid (2 TMS)	17.83	1.33
γ-Aminobutyric acid (3 TMS)	23.12	1.28
Glyceric acid (3 TMS)	20.52	1.22
Gluconic acid (6 TMS)	28.47	1.19

^a^Retention time (min)

^b^Variable importance in projection

^c^*TMS* Trimethylsilyl derivative

Consistently, malic acid, succinic acid, citric acid, and fumaric acid, all involved in TCA cycle, increased during algal growth. It is well recognized that endogenous organic acids are the source of both carbon skeleton and energy for cells and are used in the respiratory cycle and other biochemical pathways. Previous studies demonstrated that the malic acid pathway is critical for lipid accumulation (Xue et al. 2016; Red et al. 2016), γ-aminobutyric acid (GABA) was also found upregulated during growth suggesting this metabolite may play a molecular signaling role in response to various environmental stress, including oxidative stress (Bouché and Fromm 2004).

To sum up the GC–MS metabolomics data, this study provides an overview of the dynamic metabolic changes during 7 weeks on the SCCA strain *C. melkonianii*, which potentially provides screening for the selection of their biologically active natural products. Moreover, the results of the metabolite analysis in this work can be used to further explore the uses and product synthesis of this microalga, which might be relevant for different biotechnological and bioremediation applications.

### UHPLC-QTOF-MS/MS analysis

In this work, we also investigated the lipid profile of *C. melkonianii,* in the last stages of growth, using a UHPLC-QTOF-MS/MS analytical platform after lipid extraction with chloroform. Total percent composition, based on ionic abundance, of different lipids species is reported in Table 3. TAG (61.34 ± 0.60%) were the prevalent lipids followed by LDGTS, GlcADG, DGTS and SQDG. In particular, in the ESI + mode, the lipid profile, expressed as µg/mL and percent composition, is reported in Table 4. The most abundant triacylglycerols were: TAG (16:0_18:1_18:1), TAG (16:0_18:1_18:2), TAG (18:1_18:1_18:1), and TAG (18:1_18:1_18:2) with a carbon number of annotated TAG comprised between 48 and 56 with 9 as the maximum number of insaturation. We were also able to annotate different lysobetaine lipids, such as lysodiacylglyceryltrimethylhomoeserine 16:0 (LDGTS 16:0) and different betaine lipids diacylglyceryltrimethylhomoeserine with carbon number, comprised between 18 and 34 and 3 as the maximum number of insaturations. Interestingly, based on their abundance, we measured the percent levels of different glucoronosyl diacylglycerol 34:1 and 34:2 and sulfoquinovosyldiacylglycerols (SQDG): SQDG (16:0_16:0), SQDG (16:0_18:2), SQDG (16:0_18:3) and SQDG (16:0_18:1).

**Table 3:** Percent composition of the lipid classes determined both in the positive and negative ionization mode

Lipids class	Positive	Negative
	%	%
TAG	61.34 ± 0.60%	–
DAG	4.66 ± 0.03%	–
DGTS	11.55 ± 0.09%	–
LDGTS	5.29 ± 0.04%	–
SQDG	11.34 ± 0.10%	1.03 ± 0.02%
GlcADG	1.35 ± 0.01%	0.46 ± 0.03%
PG	0.20 ± 0.02%	0.27 ± 0.01%
PC	3.97 ± 0.03%	0.04 ± 0.01%
PE	0.30 ± 0.01%	–
DGDG	–	0.41 ± 0.02%
MGDG	–	0.46 ± 0.01%
Cer_ADS	–	0.41 ± 0.02%
Cer_AP	–	0.03 ± 0.01%
Cer_NDS	–	0.31 ± 0.02%
LPC	–	0.10 ± 0.01%
FA	–	95.38 ± 0.45%
FAHFA	–	1.12 ± 0.01%

**Table 4:** *C. melkonianii* lipid concentration (µg/mL) and percent composition detected in positive ionization mode

Lipids class	Sum compostion	*m/z*	ug/mL	%
TAG 48:0	16:0_16:0_16:0	824.76523	0.06 ± 0.01	0.05
TAG 48:2	14:0_16:0_18:2	820.73397	0.05 ± 0.01	0.04
TAG 49:1	15:0_16:0_18:1	836.76596	018 ± 0.06	0.14
TAG 50:1	16:0_16:0_18:1	850.78464	6.75 ± 0.25	5.14
TAG 50:2	16:0_16:0_18:2	848.76901	3.35 ± 0.30	2.55
TAG 50:3	16:0_16:0_18:3	846.75257	13.5 ± 0.12	1.03
TAG 50:4	16:0_16:3_18:1	844.73610	0.80 ± 0.01	0.61
TAG 50:5	16:0_16:3_18:2	842.72008	0.56 ± 0.01	0.43
TAG 50:6	16:0_16:3_18:3	840.70487	0.86 ± 0.01	0.65
TAG 50:7	16:1_16:3_18:3	838.68719	0.11 ± 0.01	0.08
TAG 50:9	16:3_16:3_18:3	834.65624	0.26 ± 0.01	0.20
TAG 51:2	15:0_18:1_18:1	862.78124	0.19 ± 0.01	0.14
TAG 51:3	15:0_18:1_18:2	860.76521	0.09 ± 0.01	0.07
TAG 52:1	16:0_18:0_18:1	878.81613	5.11 ± 0.15	3.88
TAG 52:2	16:0_18:1_18:1	876.80026	14.08 ± 1.02	10.71
TAG 52:3	16:0_18:1_18:2	874.78478	10.45 ± 1.09	7.94
TAG 52:4	–	872.76909	5.68 ± 0.45	4.32
TAG 52:5	16:0_18:2_18:3	870.75301	2.42 ± 0.19	1.84
TAG 52:6	–	868.73638	1.07 ± 0.09	0.81
TAG 52:7	16:3_18:1_18:3	866.72054	0.96 ± 0.01	0.73
TAG 52:8	16:3_18:2_18:3	864.70478	0.86 ± 0.01	0.65
TAG 52:9	16:3_18:3_18:3	862.68916	0.91 ± 0.01	0.69
TAG 53:2	17:0_18:1_18:1	890.81208	0.08 ± 0.01	0.06
TAG 54:1	18:0_18:0_18:1	906.84588	1.04 ± 0.09	0.79
TAG 54:2	18:0_18:1_18:1	904.83184	6.02 ± 0.22	4.58
TAG 54:3	18:1_18:1_18:1	902.81586	9.32 ± 0.88	7.09
TAG 54:4	18:1_18:1_18:2	900.80034	7.95 ± 0.79	6.05
TAG 54:5	18:1_18:2_18:2	898.78457	4.54 ± 0.21	3.45
TAG 54:6	18:1_18:2_18:3	896.76847	2.07 ± 0.12	1.58
TAG 54:7	18:2_18:2_18:3	894.75109	0.49 ± 0.02	0.37
TAG 56:3	18:1_18:2_20:0	930.84324	0.11 ± 0.01	0.09
DAG 34:1	16:0_18:1	612.55302	0.11 ± 0.01	0.08
DAG 34:2	16:0_18:2	610.53828	0.43 ± 0.04	0.32
DAG 34:3	16:0_18:3	608.52223	0.22 ± 0.02	0.17
DAG 36:2	18:1_18:1	638.56963	0.45 ± 0.02	0.34
DAG 36:4	18:2_18:2	634.53896	1.04 ± 0.10	0.79
DAG 36:5	18:2_18:3	632.52364	1.75 ± 0.16	1.33
DGTS 18:3^*^	2:0_16:3	510.33981	–	0.07
DGTS 32:2	16:0_16:2	708.57505	–	0.55
DGTS 32:3	16:0_16:3	706.55808	–	0.19
DGTS 34:1	16:0_18:1	738.62039	–	0.10
DGTS 34:2	16:0_18:2	736.60758	–	3.10
DGTS 34:3	16:0_18:3	734.59149	–	1.69
LDGTS 16:0	16:0	474.37875	–	8.32
LDGTS 18:1	18:1	500.39341	–	0.85
LDGTS 18:2	18:2	498.37849	–	1.78
SQDG 32:0	16:0_16:0	812.55478	–	3.04
SQDG 34:1	16:0_18:1	838.56789	–	0.38
SQDG 34:2	16:0_18:2	836.55358	–	1.21
SQDG 34:3	16:0_18:3	834.53768	–	1.01
GlcADG 34:1	16:0_18:1	788.58718	–	5.19
GlcADG 34:2	16:0_18:2	786.57042	–	0.66
PC 34:2	–	758.56782	2.18 ± 0.19	1.65
PC 36:2	–	786.59682	0.18 ± 0.01	0.14
PC 36:3	–	784.58165	0.17 ± 0.01	0.13
PC 36:4	18:2_18:2	782.56578	0.17 ± 0.01	0.13
PE 33:1	–	704.51890	0.04 ± 0.01	0.03

*These lipid classes were not quantitated because of the lack of commercial standards

In the ESI mode, microalga samples showed the most abundant fatty acids (Table 5) were the oleic acid (FA 18:1, C18:1omega9), linoleic acid (FA 18:2, C18:2omega6), palmitic acid (FA 16:0), and stearic acid (FA 18:0) at 29.11, 20.57, 19.38 and 17.13%, respectively. Complex lipids determined in the negative ionization mode were dominated by phosphatidylglycerol (16:1_18:2) with a percent level of 73.29% (Table 6). Noteworthy, in *C. melkonianii* samples, we were able to annotate in branched fatty acid esters of hydroxy fatty acids (FAHFA). These compounds represent a class of functional lipids called lipokines. The comparison of annotated FAHFA showed that *C. melkonianii* samples are characterized by a unique fingerprint of FAHFA, such as FAHFA (18:1_20:3), FAHFA (18:2_20:4), FAHFA (18:0_20:2), and FAHFA (18:1_18:0). FAHFAs were recently identified as a class of bioactive lipids with anti-diabetic and anti-inflammatory activities (Yore et al. 2014). Lower levels of mono- and di-galactosyldiacylglycerols (MGDG and DGDG, respectively), ceramide alpha-hydroxy fatty acid-dihydrosphingosines (Cer_ADS), ceramide non-hydroxyfatty acid-hihydrosphingosine (Cer_NDS), ceramide alpha-hydroxy fatty acid-phytospingosine (Cer_AP) and lysophatidylcholine LPC (18:2_0:0) were also found. The ratio between the two major galactolipids, MGDG and DGDG, is variable in microalgae since it is strongly influenced by responses to environmental and nutritional cues (Khozin-goldberg 2016). Higher-plant and microalgae chloroplast are made of four characteristic lipids, PG, MGDG, DGDG, and SQDG (Shimojima 2011; Boudière et al. 2014; Da Costa et al. 2016). The latter is sulfur-containing anionic glycerolipid component of photosynthetic membrane lipids, UDP-sulfoquinovose synthase (SQD1) and SQDG synthase (SQD2) which is responsible of the SQDG biosynthesis (Shimojima 2011). The synthesis of SQDG start from the entering of sulfates into the microalgae chloroplast. Sulfates are first transformed to adenosine 5’-phosphosulfate by iron − sulfur flavoenzyme adenosine-5 ‘-phosphosulfate (APS) and then to sulphites. The enzymatic complex UDP-sulfoquinovose synthase and ferredoxin-dependent glutamate synthase catalyze the reaction of sulphites with UDP-glucose to form UDP-sulfoquinovose (UDP-SQ). SQDG synthase (SQD2) catalyzes the reaction of UDP-SQ with DAG to form SQDG (Shimojima 2011). In the environment, this pathway may allow the microalgae *C*.* melkonianii* to efficiently cope with high levels of sulfates measured in the Rio Irvi, i.e., 3694 mg/L. In summary, in this work, we reported the primary metabolic changes during growth and the lipidomic characterization of the extremophile *C. melkionanii* SCCA48.

**Table 5:** Percent fatty acid (FA) composition determined in the negative ionization mode

Common name	Sum composition	*m/z*	Rt (min)^a^	%
Palmitic acid	16:0	255.23387	2.0698	19.38
Palmitoleic acid	16:1	253.21827	1.6502	1.22
Hexadecadienoic acid	16:2	251.20281	1.3565	3.35
Margaric acid	17:0	269.24921	2.4359	0.33
Margoleic acid	17:1	267.23349	1.9433	0.31
Stearic acid	18:0	283.26501	3.0121	17.16
Oleic acid	18:1	281.24968	2.2224	29.11
Linoleic acid	18:2	279.23403	1.7823	20.57
Linolenic acid	18:3	277.21834	1.4918	5.85
Nonadecylic acid	19:0	297.28014	3.3113	0.03
	19:1	295.26466	2.5800	0.22
Arachidic acid	20:0	311.29606	4.0284	1.40
Eicosanoic acid	20:1	309.28070	2.9874	0.73
Heneicosylic acid	21:0	325.31179	4.4512	0.09
Docosanoic acid	22:0	339.32732	5.1328	0.24
Erucic acid	22:1	337.31152	3.9568	0.02

^a^Retention time (minutes)

**Table 6:** Percent composition (based on abundances) of lipids detected in the negative ionization mode

Lipids class	Sum composition	*m/z*	Rt (min)^a^	%
PG 34:3	16:1_18:2	743.48774	5.3165	6.48
PG 34:4	16:1_18:3	741.47193	4.6088	2.27
DGDG 34:1	16:0_18:1	977.64151	7.6360	0.81
DGDG 34:2	16:0_18:2	975.6266	6.6618	4.67
DGDG 34:3	16:1_18:2	973.61056	5.8207	0.75
DGDG 34:4	16:2_18:2	971.59524	4.9046	3.89
DGDG 34:5	16:2_18:3	969.57918	4.2101	1.22
DGDG 34:6	16:3_18:3	967.56404	3.6213	1.23
DGDG 36:4	18:2_18:2	999.62629	5.9381	1.35
MGDG 34:1	16:0_18:1	815.58983	9.0918	2.47
MGDG 34:2	16:0_18:2	813.57385	8.0436	1.85
MGDG 34:4	16:2_18:2	809.5425	6.0703	2.23
MGDG 34:5	16:2_18:3	807.52694	5.2512	1.15
MGDG 34:6	16:3_18:3	805.51176	4.5245	1.69
MGDG 35:1	18:0_17:1	829.60524	9.6855	1.12
MGDG 36:3	18:1_18:2	839.5894	8.2732	1.29
MGDG 37:2	18:1_19:1	841.60538	9.3117	3.03
Cer_ADS d42:0	d42:0	726.6628	13.5737	10.73
Cer_ADS d40:0	d40:0	698.63117	12.2328	3.36
Cer_ADS d43:0	d43:0	740.67783	14.0945	1.27
Cer_ADS d41:0	d41:0	712.64738	12.9357	6.82
Cer_ADS d44:0	d44:0	754.69355	14.5210	1.63
Cer_AP t41:0	t41:0	668.62062	12.2178	0.96
Cer_NDS d38:0	d38:0	654.60507	12.0614	1.65
Cer_NDS d34:0	d34:0	598.5427	9.1161	2.03
Cer_NDS d36:0	d36:0	626.57352	10.6070	5.18
Cer_NDS d42:0	d42:0	710.66756	14.4443	1.05
LPC 18:2	18:2/0:0	578.34718	1.3189	3.32
FAHFA 38:2	18:0_20:2	589.51898	2.8522	11.44
FAHFA 36:1	18:1_18:0	563.50537	2.2196	1.43
FAHFA 38:4	18:1_20:3	585.48810	2.2235	23.17
FAHFA 38:6	18:2_20:4	581.45649	1.7825	15.97

The changes of cellular polar metabolites during growth reported in this work may be potentially useful to control and adapt biofuel synthesis in *Trebouxiophyceae*. Besides, FA (i.e., oleic acid, linoleic acid, palmitic acid and stearic acid), this strain showed high levels of the following lipid classes: TAGs, LSGTSs, SQSGs and DGTS. Our results give a basic biochemical, metabolomic and lipidomic, snapshot of this algae to be used for the engineering of biological phycoremediation filters able to reduce freshwaters pollution as open raceway pond or closed photobioreactor. Furthermore, the in-depth lipidomic characterization of this strains opens the road to produce isotopically labeled standards of lipids to be used in the field of biochemistry.

## Abbreviations

TAG: Triacylglycerol
DAG: Diacylglycerol
PG: Phosphatidylglycerol
PC: Phosphatidylcholine
PE: Phosphatidylethanolamine
LPC: Lysophophatidylcholine
SM: Sphingomyelin
DGDG: Digalactosyldiacylglycerol
DGTS: Diacylgyceryltrimethylhomoserine
LDGTS: Lysodiacylglyceryltrimethylhomoserine
MGDG: Monogalactosyldiacylglycerol
SQDG: Sulfoquinovosyl diacylglycerol
GlcADG: Glucoronosyldiacylglycerol
Cer_ADS: Ceramide alpha-hydroxy fatty acid-dihydrosphingosine
Cer_AP: Ceramide alpha-hydroxy fatty acid-phytospingosine
Cer_NDS: Ceramide non-hydroxyfatty acid-dihydrosphingosine
FA: Free fatty acid
FAHFA: Fatty acid ester of hydroxyl fatty acid
